# Dataset of Trans-Resveratrol on diabetes-induced abnormal spermatogenesis, poly (ADP-ribose) polymerase-1 (PARP1) expression in intra-testicular blood vessels, and stage-dependent expression of PARP1 and Sirtuin 1 in the rat testis

**DOI:** 10.1016/j.dib.2016.11.095

**Published:** 2016-12-06

**Authors:** Ala׳ Abdelali, Maie Al-Bader, Narayana Kilarkaje

**Affiliations:** a Department of Anatomy, Faculty of Medicine, Kuwait University, Kuwait; b Department of Physiology, Faculty of Medicine, Kuwait University, Kuwait

**Keywords:** Apoptosis, DNA damage repair, Hyperglycemia, Male germ cells

## Abstract

This article contains data related to the article “Effects of Trans-Resveratrol on hyperglycemia-induced abnormal spermatogenesis, DNA damage and alterations in poly (ADP-ribose) polymerase signaling in rat testis” (A. Abdelali, M. Al-Bader, N. Kilarkaje, 2016) [1]. The data are related to Resveratrol on diabetes-induced changes in blood glucose levels, body weights of rats, sperm count and motility, expression of poly (ADP-ribose) polymerase-1 (PARP1) in Leydig cells and in intratesticular blood vessels, and stage-dependent expression of PARP1 and Sirtuin 1 (SirT1) in the rat testis. In this experiment, the data were obtained from control, Resveratrol-treated, diabetic and Resveratrol-treated diabetic rats on day 42 after the induction of diabetes. Resveratrol treatment for a group each of normal and diabetic rats started on day 22 and extended up to day 42. The sperm parameters were conducted in samples obtained from the epididymis. The expression of proteins was evaluated by immunohistochemistry by using specific primary antibodies. The data are presented in the form of figures and significance of them has been given in the research article [1].

**Specifications Table**

**Table:** 

Subject area	*Biology*
More specific subject area	*Resveratrol effects on diabetes-induced changes in spermatogenesis and PARP signaling*
Type of data	*Figures*
How data was acquired	1. *For the estimation of glucose level, the serum was used and the glucose content was measured by using a glucometer* 2. *For quantification of sperm motility and sperm count, the sperm from the tails of epididymides were collected and analyzed as described before* [2]. 3. *For the analysis of protein expression, specific primary antibodies and appropriate secondary antibodies were used and immunohistochemical analysis was carried out. Expression of PARP1 was evaluated in Leydig cells and in intra-testicular blood vessels. Stage-dependent expression of PARP1 and SirT1 was also analyzed* [2].
Data format	*Analyzed*
Experimental factors	*Adult 13–15 week-old male Wistar rats were used. Diabetes was induced by Streptozotocin injection. The rats were diabetic for 42 days. Resveratrol (5 mg/kg/day) was given to diabetic rats starting from day 22 up to day 42, on which day they were killed for sample collection (testicular and epididymal).*
Experimental features	*Experimental animals were segregated into 1) control, 2) Resveratrol-treated (5 mg/kg; ip), 3) Streptozotocin (55 mg/kg; ip)-induced diabetic, and 4) diabetic+ Resveratrol-treated groups.*
Data source location	*n/a*
Data accessibility	*The data are included with this article.*

**Value of the data**

• These data may be useful to revisit the hitherto widely believed notion that Resveratrol inhibits hyperglycemia.
• The data about Resveratrol on diabetes-induced changes in sperm motility and sperm count are promising and may be useful for alleviating these abnormalities of diabetes in patients.
• Thus far, diabetes is known to cause adversities in the testis. This data set provides proof for the fact that PARP1 expression in intra-testicular blood vessels may have contributory roles for the onset of testicular damage.
• PARP1 expression in the testis is seminiferous epithelial stage-dependent. This will help researchers to find out which germ cell types are susceptible to changes in PARP1 expression because of hyperglycemia and Resveratrol.
• The data set provides evidence in favor of stage-independent expression of SirT1 in the testis. This data set may assist researchers to further understand which germ cell types are important in providing antioxidant synthesis or stimulation in the testis.

## 1 Data

**Fig. 1:**
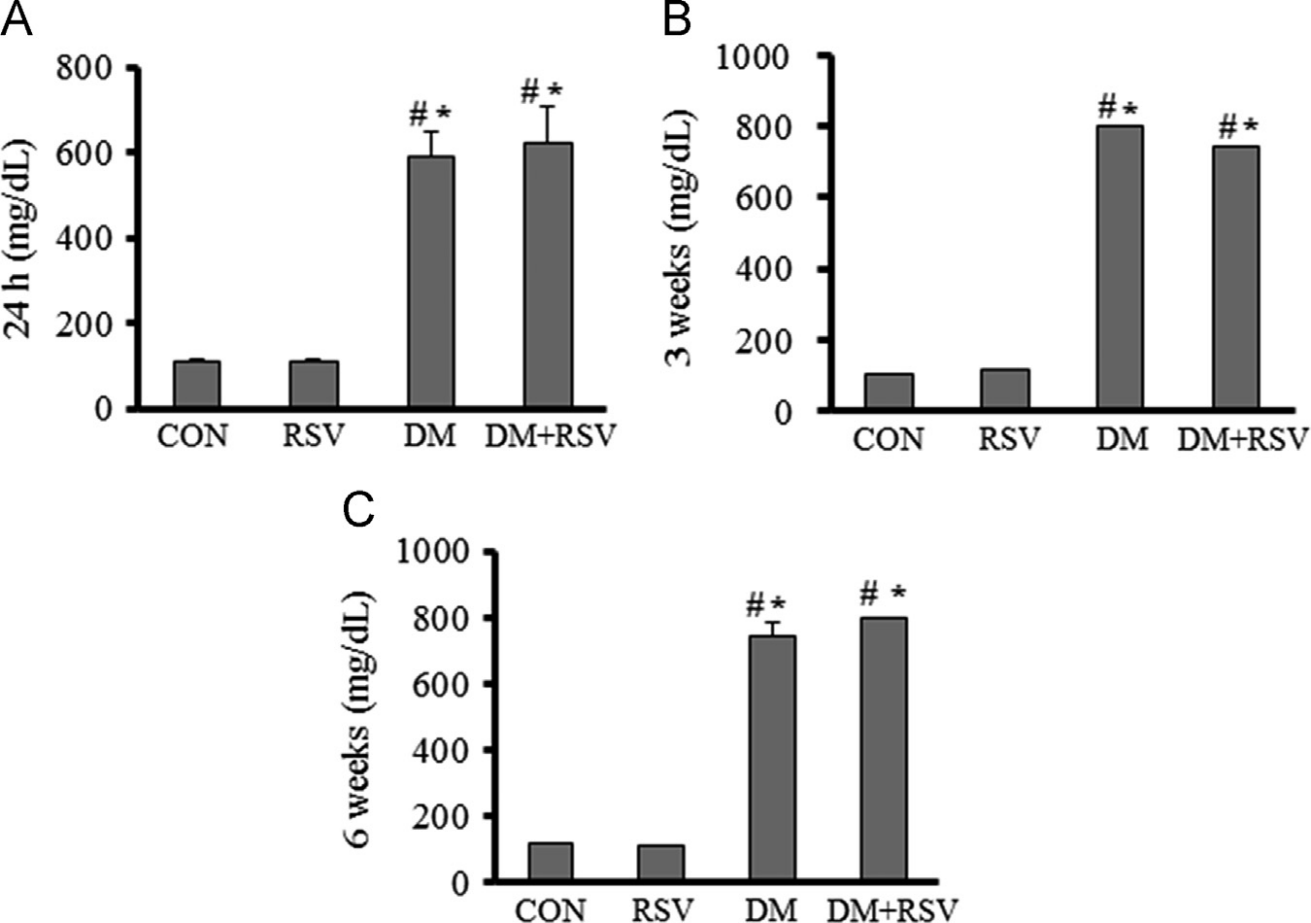
Effects of Resveratrol (RSV) on hyperglycemia in rats at 24 h (A), 3 weeks (B) and 6 weeks (C) after the induction of diabetes (DM). Data are represented as mean+S.E.M for each group (*n*=6). *P<0.05, control (CON) versus experimental groups; ^#^*P*<0.05, RSV versus other experimental groups.

**Fig. 2:**
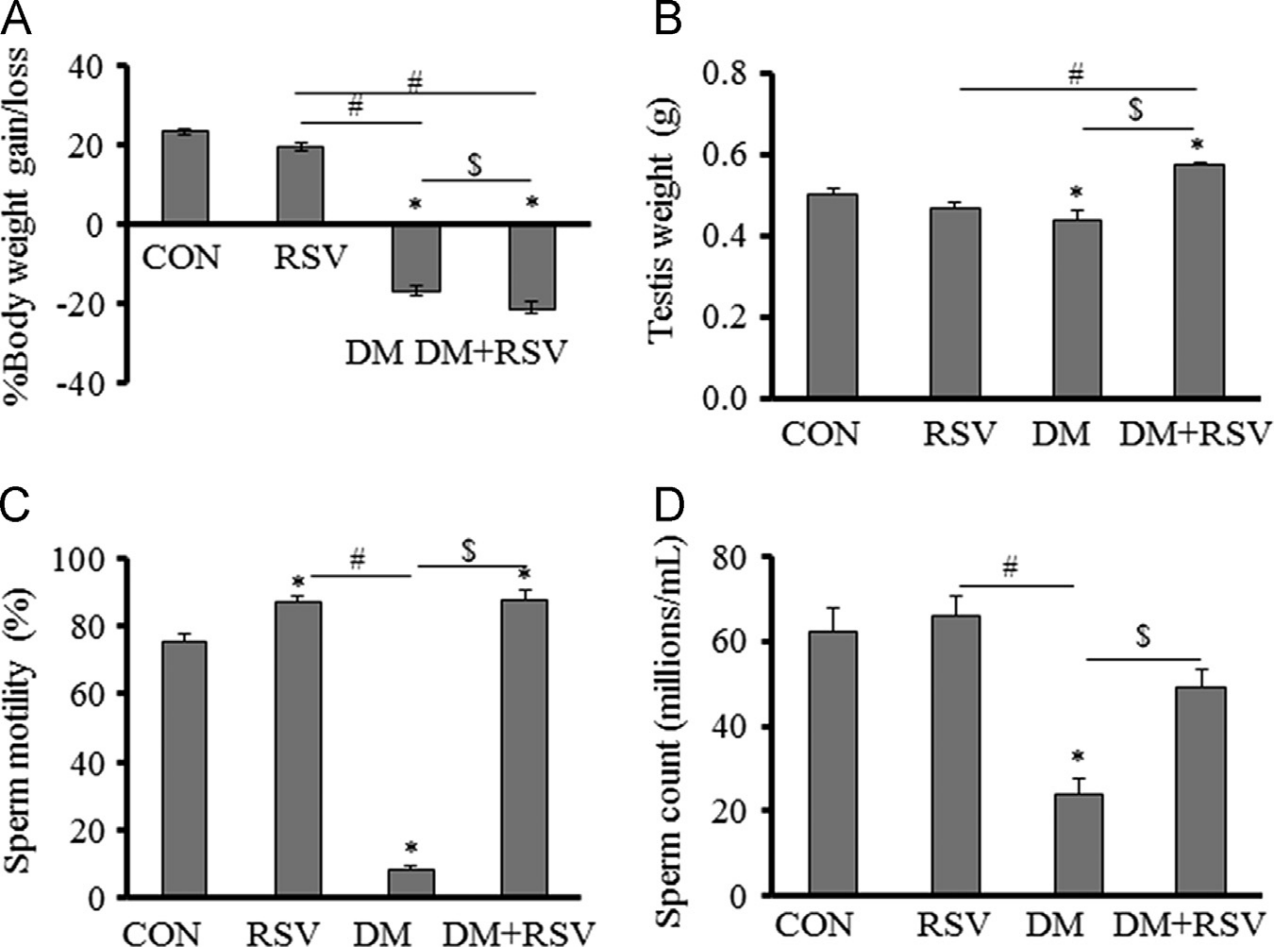
Effects of Resveratrol (RSV) on diabetes (DM)-induced effects on A) % body weight gain/loss, B) testis weight, C) sperm motility (%), and D) sperm count (millions/ml) in rats. Data are represented as mean+S.E.M for each group (*n*=6). *P<0.05, control (CON) versus experimental groups; ^#^*P*<0.05, RSV versus other experimental groups; ^$^*P*<0.05, DM versus DM+RSV.

**Fig. 3:**
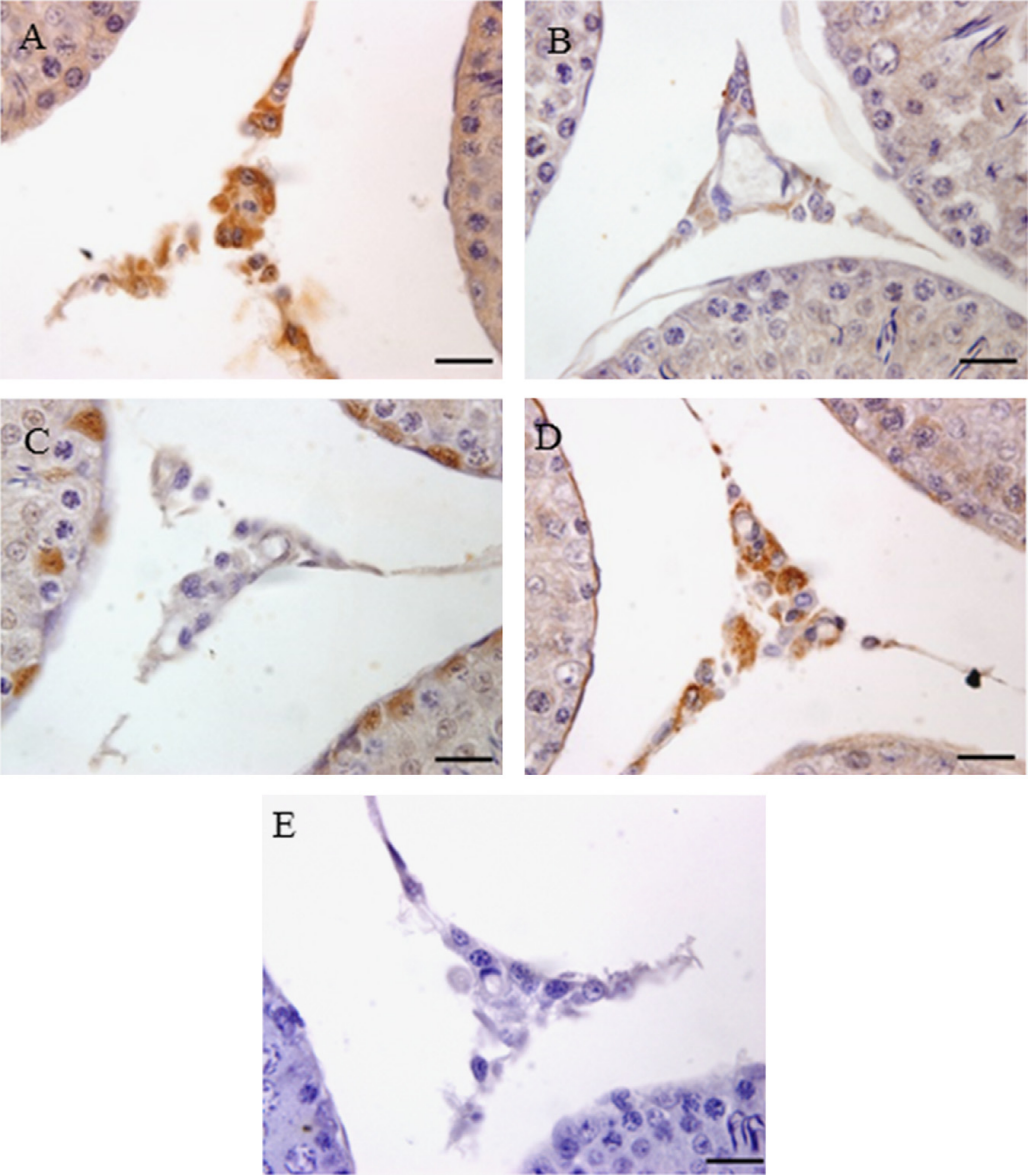
Immunohistochemistry photomicrographs showing localization of PARP1 in Leydig cells in the testis. (A) Control, (B) Resveratrol, (C) Diabetes, (D) Diabetes + Resveratrol and (E) negative control in which PARP1 primary antibody was not used. Photomicrographs were counterstained with Mayer׳s hematoxylin; magnification, 1000X (scale bar=20 μm).

**Fig. 4:**
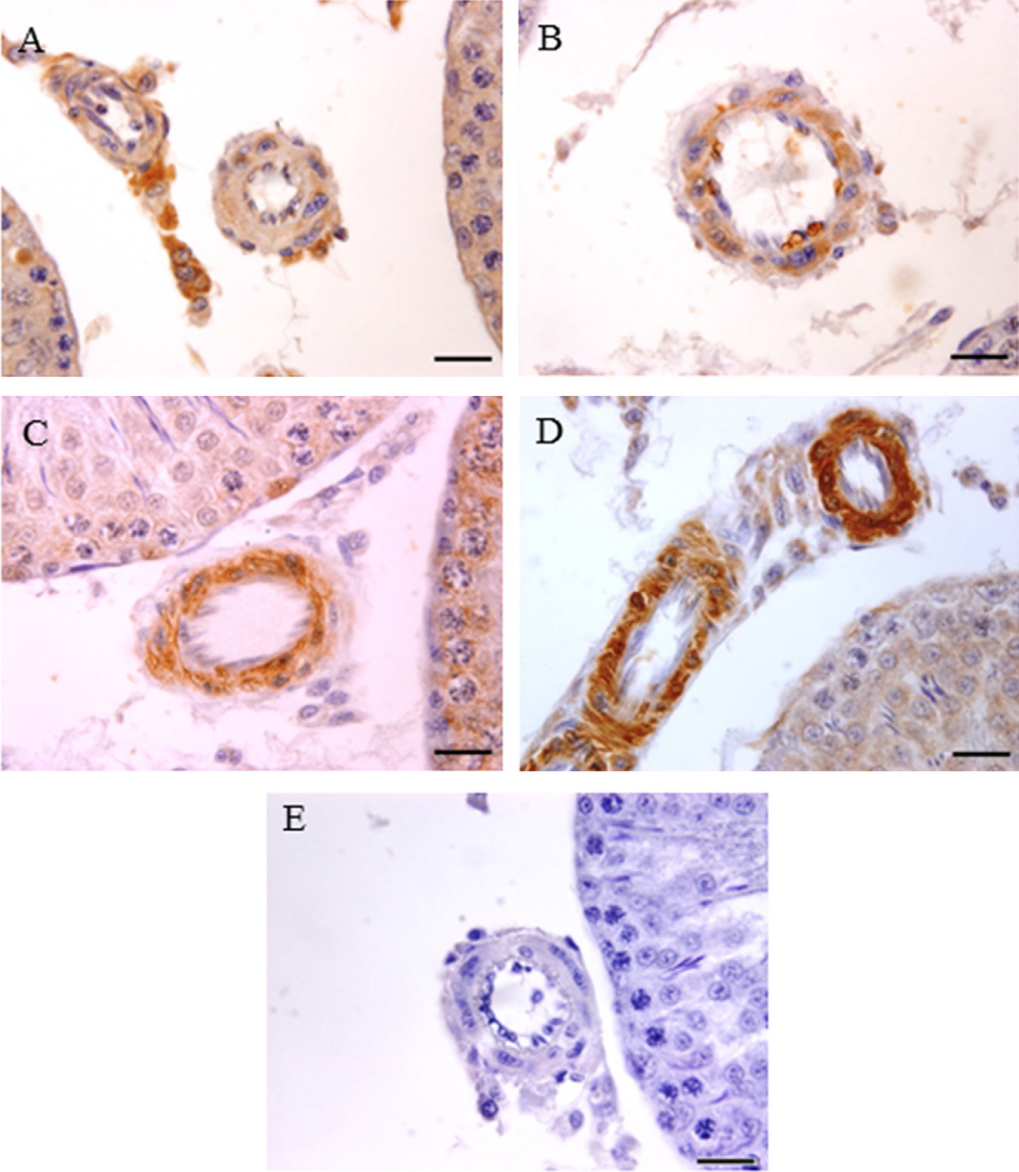
Immunohistochemistry photomicrographs showing localization of PARP1 in blood vessels in the testis. (A) Control, (B) Resveratrol, (C) Diabetes, (D) Diabetes + Resveratrol and (E) negative control in which PARP1 primary antibody was not used. Photomicrographs were counterstained with Mayer׳s hematoxylin; magnification, 1000X (scale bar=20 μm).

**Fig. 5:**
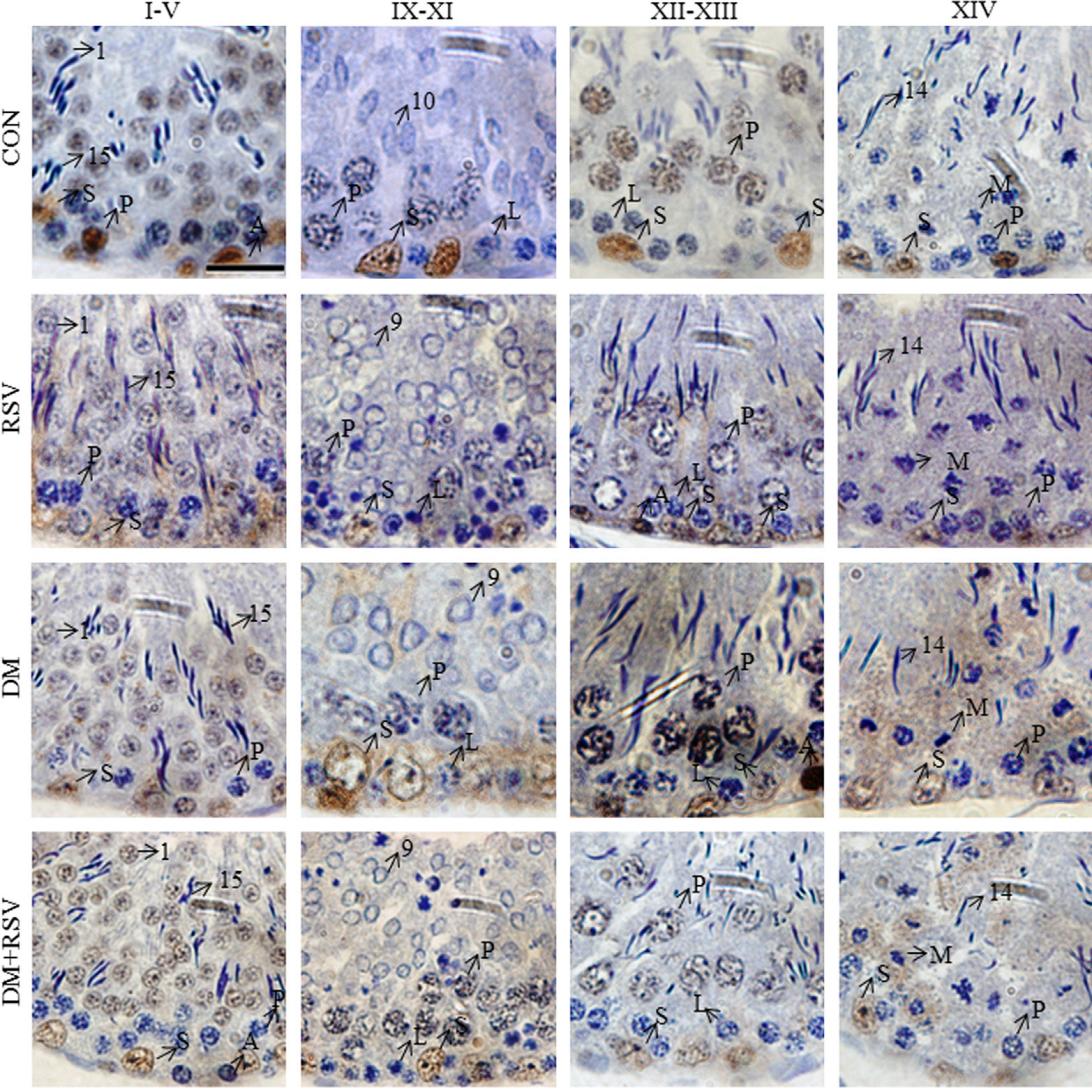
Representative photomicrographs of PARP1 immunohistochemistry showing seminiferous epithelial stage-dependent expression of the protein. Photomicrographs of control (CON), Resveratrol (RSV), diabetes (DM) and diabetes + Resveratrol (DM+RSV)-treated rat testes (*n*=3). 1000X magnification (scale bar=20 μm). A, spermatogonia; M, meiotic figures; L, leptotene spermatocytes; P, pachytene spermatocytes; S, Sertoli nuclei; 1–19, spermatid steps; Counterstained with Mayer׳s hematoxylin.

**Fig. 6:**
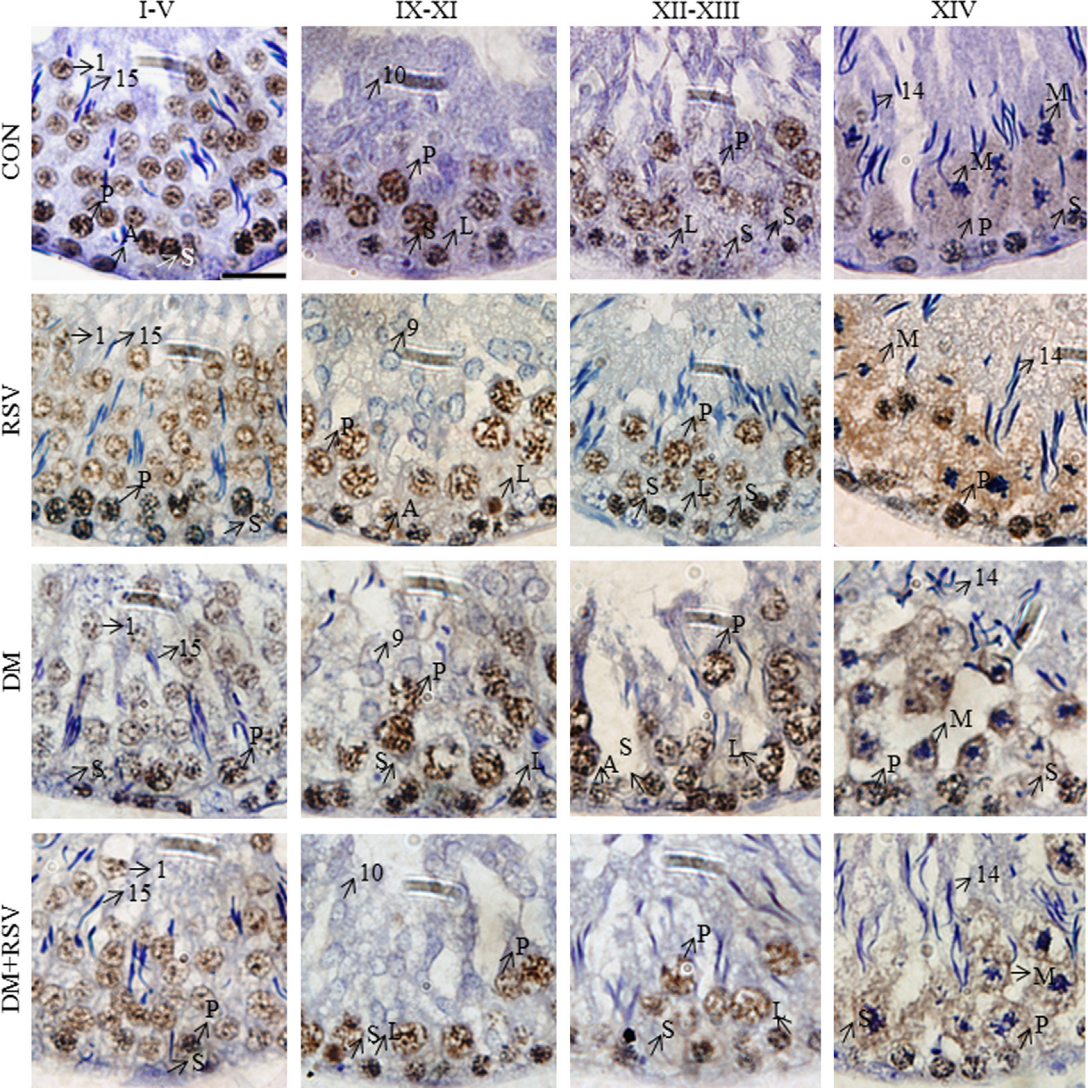
The representative photomicrographs of SirT1 immunohistochemistry showing stage-independent expression of the protein. Photomicrographs of control (CON), Resveratrol (RSV), diabetes (DM) and diabetes + Resveratrol (DM+RSV)-treated rat testes (*n*=3). 1000X magnification (scale bar=20 μm). A, spermatogonia; M, meiotic figures; L, leptotene spermatocytes; P, pachytene spermatocytes; S, Sertoli nuclei; 1–19, spermatid steps; Counterstained with Mayer׳s hematoxylin.

The data set presents blood glucose levels, changes in body weight and testis weight, sperm count and motility, and expression of PARP1 and SirT1 in the testis. Fig. 1, Fig. 2 indicate blood glucose levels, body weight, and testis weight and sperm parameters. Fig. 3, Fig. 4 are a visual evidence for the changes due to diabetes and Resveratrol in expression of PARP1 in Leydig cells and in intra-testicular blood vessel smooth muscle cells. Fig. 5, Fig. 6 present data supporting the seminiferous epithelial stage-dependent expression of PARP1 and stage-independent expression of SirT1 in the testis.

## 2 Experimental design, materials and methods

Diabetes was induced in adult male Wistar rats by a single injection of Streptozotocin (50 mg/kg) and hyperglycemia was confirmed by measuring blood glucose levels at 24 h after the injection and sustained hyperglycemia was again confirmed at 3 weeks and 6 weeks after the injection. One diabetic group of rats was injected Trans-Resveratrol intraperitoneally (cat#501-36-0; Cayman Chemicals, USA; purity ≥98%) 5 days/week, once daily starting from day 22 to 42 after the confirmation of hyperglycemia. The rats were sacrificed on 42 day and the reproductive organs were removed. All animal experiments were conducted in accordance with the ARRIVE guidelines.

The epididymides were removed and their filtrate were prepared and used for estimating the sperm parameters. The number of motile sperm was counted by observing the filtrate under a light microscope and expressed as percentage incidence. The number of sperm in millions was counted by using a Neaubar׳s counting chamber [2], [3].

Testes were fixed in Bouin׳s fluid and processed for paraffin embedding. Five µm thick sections were cut and used for immunohistochemistry. This method was carried out by using specific primary antibodies against PARP1 and SirT1 as per the procedure described before [1], [3]. The data were obtained by observing the tissue sections under a light microscope. The expression of PARP1 was evaluated in Leydig cells and intra-testicular blood vessels. Stage-dependent expression of PARP1 and SirT1 was evaluated in seminiferous tubules. Representative photographs were taken and used to present the data.

## 3 Data analysis

The data were statistically analyzed for significance by using one way analysis of variance and Fisher׳s least square difference test with significance level set at *P*<0.05.
